# Chitosan-functionalized lipid-polymer hybrid nanoparticles for oral delivery of silymarin and enhanced lipid-lowering effect in NAFLD

**DOI:** 10.1186/s12951-018-0391-9

**Published:** 2018-09-04

**Authors:** Jun Liang, Ying Liu, Jinguang Liu, Zhe Li, Qiangyuan Fan, Zifei Jiang, Fei Yan, Zhi Wang, Peiwen Huang, Nianping Feng

**Affiliations:** School of Pharmacy, Shanghai University of Traditional Chinese Medicine, Shanghai, 201203 China

**Keywords:** Chitosan-functioned lipid-polymer hybrid nanoparticle, Silymarin, Oral bioavailability, Lipid-lowering effect, Non-alcoholic fatty liver disease

## Abstract

**Background:**

Non-alcoholic fatty liver disease (NAFLD) is a chronic disease that causes excessive hepatic lipid accumulation. Reducing hepatic lipid deposition is a key issue in treatment and inhibition of NAFLD evolution. Silymarin is a potent hepatoprotective agent; however, it has low oral bioavailability due to its poor aqueous solubility and low membrane permeability. Unfortunately, few studies have addressed the development of convenient oral nanocarriers that can efficiently deliver silymarin to the liver and enhance its lipid-lowering effect. We designed silymarin-loaded lipid polymer hybrid nanoparticles containing chitosan (CS-LPNs) to improve silymarin bioavailability and evaluated their lipid-lowering effect in adiponutrin/patatin-like phospholipase-3 I148M transgenic mice, an NAFLD model.

**Results:**

Compared to chitosan-free nanoparticles, CS-LPNs showed 1.92-fold higher uptake by fatty liver cells. Additionally, CS-LPNs significantly reduced TG levels in fatty liver cells in an in vitro lipid deposition assay, suggesting their potential lipid-lowering effects. The oral bioavailability of silymarin from CS-LPNs was 14.38-fold higher than that from suspensions in rats. Moreover, compared with chitosan-free nanoparticles, CS-LPNs effectively reduced blood lipid levels (TG), improved liver function (AST and ALT), and reduced lipid accumulation in the livers of mice in vivo. Reduced macrovesicular steatosis in pathological tissue after CS-LPN treatment indicated their protective effect against liver steatosis in NAFLD.

**Conclusions:**

CS-LPNs enhanced oral delivery of silymarin and exhibited a desirable lipid-lowering effect in a mouse model. These findings suggest that CS-LPNs may be a promising oral nanocarrier for NAFLD therapeutics.

**Electronic supplementary material:**

The online version of this article (10.1186/s12951-018-0391-9) contains supplementary material, which is available to authorized users.

## Background

Non-alcoholic fatty liver disease (NAFLD) is becoming the most common chronic liver disease worldwide [1]. In recent years, changes in lifestyle have led to a rapid increase in the incidence of NAFLD. The disease results from interactions among multiple factors, including diet, the environment, metabolism, and genetics [2]. It is characterized by excessive lipid accumulation leading to hepatic disorders such as steatohepatitis, fibrosis, and cirrhosis. As a multifactorial disease, NAFLD often coexists with insulin resistance syndrome or metabolic syndromes such as obesity, diabetes mellitus, hyperlipidemia, and hypertension [3]. Comprehensive lifestyle modifications such as dietary adjustment and weight loss have been reported to be beneficial in NAFLD treatment [4]. However, the lifestyle modifications need to be maintained.

**Fig. 1:**
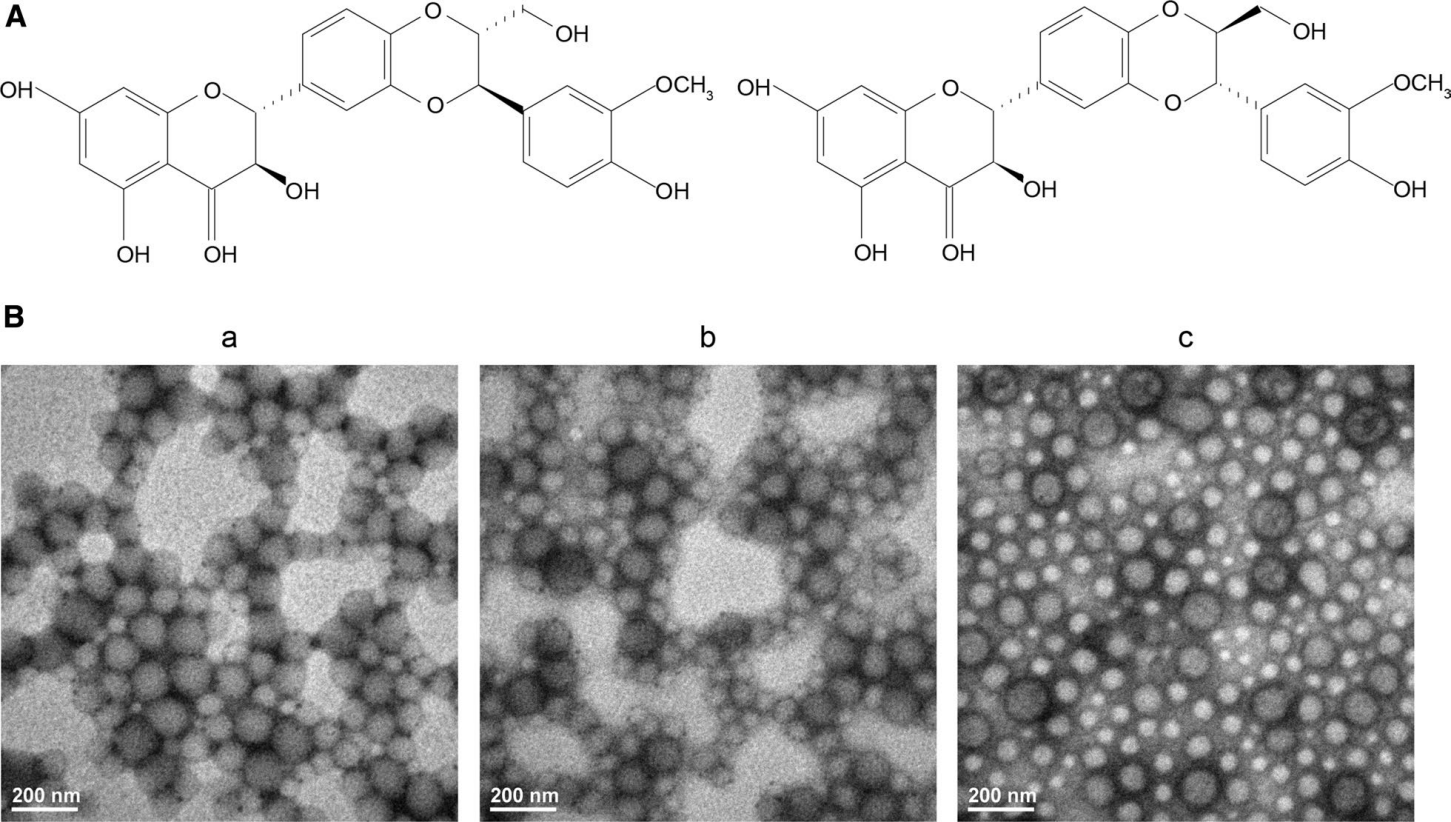
**A** Chemical structures of silybin **A** (left) and silybin **B** (right); **B** transmission electron microscopy images of (a) lipid-polymer hybrid nanoparticles (LPNs), (b) silymarin-loaded LPNs (S-LPNs), and (c) chitosan-modified S-LPNs (CS-LPNs)

Silymarin is a mixture of flavonoid compounds extracted from the seeds of *Silybum marianum* (L.) Gaertn. It is commonly used worldwide to treat fatty liver, liver injury, and hepatitis. It is regarded as one of the most promising agents for NAFLD treatment due to its remarkable hepatoprotective, antioxidant, anti-inflammatory, anti-tumor, and hypolipidemic effects [5–8]. Its mechanism of action involves cell membrane protection against radical-induced damage, simulation of polymerase and RNA transcription, and regulation of some cell signaling pathways and biological axes such as the insulin receptor substrate 1/phosphatidylinositide 3-kinase/Akt pathway and the nicotinamide adenine dinucleotide/sirtuin 1 axis, respectively [9, 10]. The active ingredients in silymarin include silybin (Fig. 1A), isosilybin, silychristin, and silydianin. The poor water solubility of silymarin limits its oral absorption (23–47%) [11] and bioavailability (0.73%) [12], thereby hindering its clinical application. Improvement of the oral bioavailability of silymarin to enhance its therapeutic efficiency is still challenging. Although various dosage forms and nanocarriers have been designed to improve silymarin bioavailability, little information is available on the potential of these nanocarriers to improve the lipid-lowering efficiency of silymarin as a hepatoprotectant.

Lipid-polymer hybrid nanoparticles (LPNs) were recently developed from liposomes and polymer nanoparticles. They possess a shell-core structure consisting of a polymer core and a phospholipid shell [13]. The former provides a stable structure that can encapsulate both water-soluble and poorly soluble drugs [14], whereas the latter contributes to increasing encapsulation efficiency [15], facilitating surface modification, and preventing rapid release of water-soluble drugs [16]. LPNs can be freeze-dried to improve their stability [17]. Furthermore, their production is expected to become industrialized. Therefore, LPNs have great potential to be effective drug carriers. Chitosan is a natural cationic aminopolysaccharide and an ideal biological material because it is biocompatible, biodegradable, and nontoxic [18, 19]. The amino group in chitosan can be protonated in weak acid conditions to become positively charged, allowing chitosan to adsorb mucin on the intestinal mucosa by electrostatic interactions. Thus, chitosan has desirable mucoadhesive properties. Additionally, it can open tight junctions between mucosal epithelial cells in the small intestine, facilitating the passage of drugs into the bloodstream [20]. Chitosan also has various biological effects, such as immunoregulatory, anti-tumor, antioxidant, and lipid-lowering effects [21]. Sumiyoshi and Kimura [22] found that low-molecular-weight chitosan reduces liver triacylglycerol and cholesterol levels in mice that are fed a high-fat diet (HFD). Moreover, it has been shown that chitosan inhibits weight gain in obese animals and significantly reduces blood lipid levels in animal models of hyperlipidemia [23, 24]. Therefore, in this study, chitosan was expected to enhance the oral absorption, as well as the hepatoprotective and lipid-lowering effects, of silymarin.

We prepared chitosan-modified, silymarin-loaded LPNs (CS-LPNs) to enhance the oral bioavailability of silymarin and improve its lipid-lowering efficacy for NAFLD treatment. Adiponutrin/patatin-like phospholipase-3 (PNPLA3) I148M transgenic mice were used as a model of NAFLD [25] to investigate the lipid-lowering effect of CS-LPNs.

## Results

### Particle size and morphology of CS-LPNs

A modified nanoprecipitation technique [13] that enables easy nanoparticle formation and a high loading of hydrophobic drugs was used to prepare the LPNs. The particle size and zeta potential of the nanoparticles are shown in Table 1 and Additional file 1: Figure S1. The LPNs had a desirable average particle size of 125.8 nm and a narrow size distribution. Additionally, the zeta potential, which suggests the possibility of obtaining suitable nanoparticles for oral administration after chitosan coating, was appropriate. Chitosan coating increased the particle size of CS-LPNs to 286.5 nm without significantly influencing encapsulation efficiency and drug loading. In addition, due to the chitosan coating, the zeta potential of the nanoparticles changed from negative values (for LPNs) to positive values (for CS-LPNs).

**Table 1:** Particle size and zeta potential of silymarin-loaded lipid-polymer hybrid nanoparticles (S-LPNs) and chitosan-modified S-LPNs (CS-LPNs) (n = 3)

Formulation	Particle size (nm)	Polydispersity index	Zeta potential (mV)	Encapsulation efficiency (%)
S-LPN	125.8 ± 7.8	0.142 ± 0.023	− 43.1 ± 8.4	97.39 ± 0.01
CS-LPN	286.5 ± 23.8	0.226 ± 0.008	45.3 ± 8.9	97.05 ± 0.01

Both LPNs and CS-LPNs were round and regular in shape as shown in Fig. 1B. The influence of chitosan coating on particle size was also confirmed by transmission electron microscopy (TEM) images. It should be noted that the difference in size measurements might possibly be due to the different methods used. The results obtained from dynamic light scattering refer to the hydrodynamic diameter of the particles or any coagulate, including hydrodynamic layers surrounding the surface of nanoparticles composed of chitosan. However, dehydration might occur due to the drying process during TEM sample preparation.

**Fig. 2:**
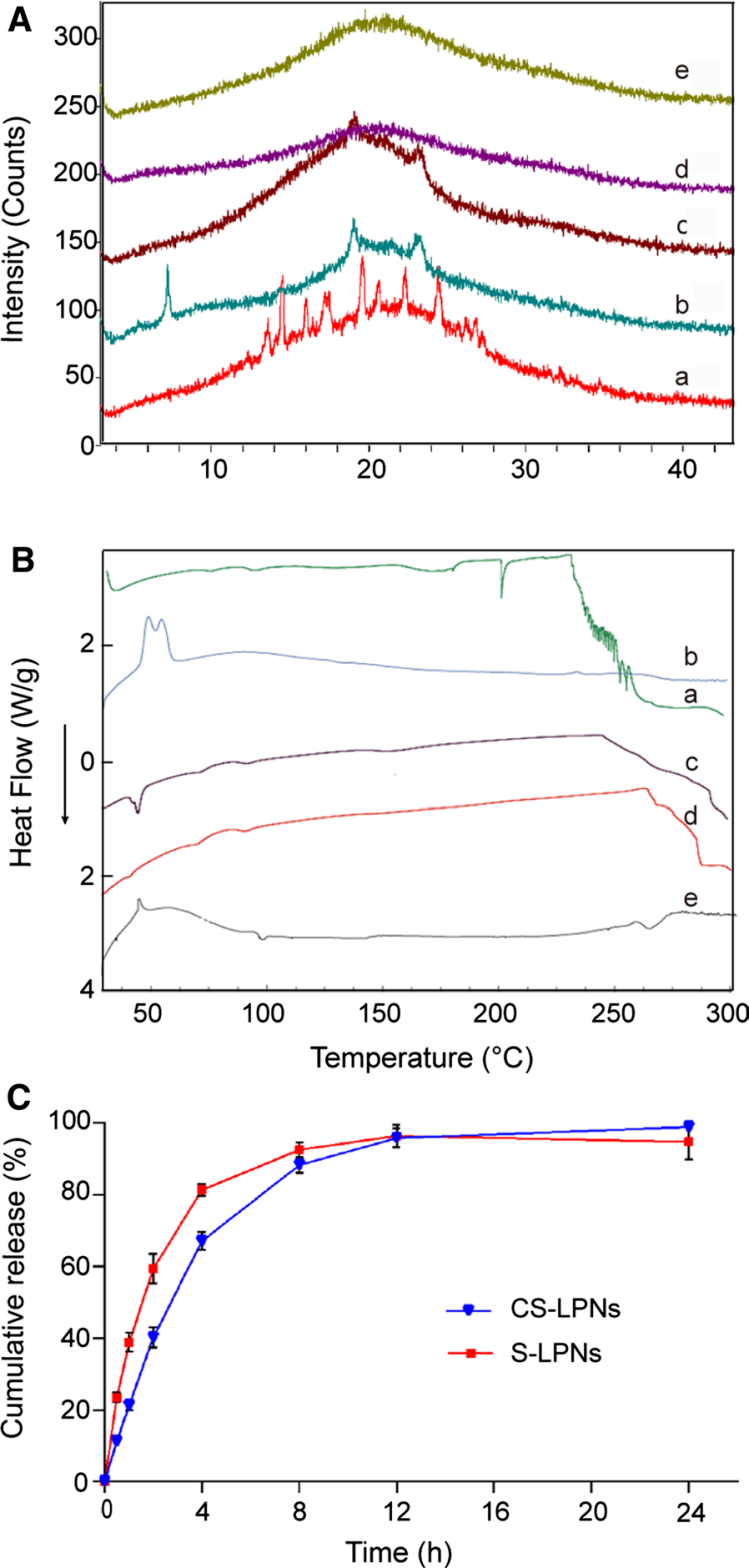
**A** X-ray powder diffraction patterns for (a) pure silymarin, (b) physical mixture, (c) lipid-polymer hybrid nanoparticles (LPNs), (d) silymarin-loaded LPNs (S-LPNs), and (e) chitosan-modified S-LPNs (CS-LPNs). **B** DSC curves of (a) raw silymarin, (b) physical mixture, (c) blank LPNs, (d) S-LPNs, and (e) CS-LPNs. **C** In vitro release profiles. Phosphate-buffered saline (pH 7.4) (containing 2% Tween 80) was used as the release medium. Data are presented as the mean ± standard deviation (n = 3)

XRD was performed to evaluate the encapsulation of silymarin in the nanoparticles by studying crystalline changes. The XRD patterns (Fig. 2A) showed that pure silymarin (a) exhibited several sharp characteristic peaks, indicating that silymarin was mainly in the crystalline form. In addition, the intensities of crystal diffraction peaks were strong. The physical mixture (b) exhibited partial diffraction peaks of silymarin crystals; however, the intensities of the characteristic peaks were weaker, suggesting that the crystalline properties of silymarin changed partially during the physical mixing with the polymer or phospholipids. Compared to the pattern for pure silymarin, the patterns for S-LPNs and CS-LPNs did not show crystal diffraction peaks of silymarin, indicating that silymarin was dispersed within the nanoparticles in its molecular form. Therefore, both preparations exhibited amorphous characteristics. To further determine the physical state of silymarin in the nanoparticles based on thermal characterization, DSC analysis was performed. Figure 2B shows the DSC curves of raw silymarin, physical mixture, blank LPNs, S-LPNs, and CS-LPNs. Compared with that for raw silymarin, the characteristic peaks of silymarin disappeared for both S-LPNs and CS-LPNs, indicating silymarin in S-LPNs and CS-LPNs was in an amorphous form, which was in accordance with the XRD results. The changes in crystalline characteristics as well as thermal changes confirm the successful encapsulation of silymarin in CS-LPNs. Moreover, a molecular dispersion of silymarin is beneficial for improved dissolution, intestinal absorption and thus enhanced bioavailability.

### In vitro drug release

The in vitro release profile (Fig. 2C) showed that CS-LPNs and S-LPNs exhibited a burst release within the first 2 h, possibly due to the dispersion of some drug on the surface of the nanoparticles. The drug release rates from both CS-LPNs and S-LPNs was higher than 95% at 12 h. Drug release from CS-LPNs was slightly lower; however, there was no significant difference in drug release between the two preparations according to the F2 similarity factor.

### Cellular uptake of the nanoparticles

**Fig. 3:**
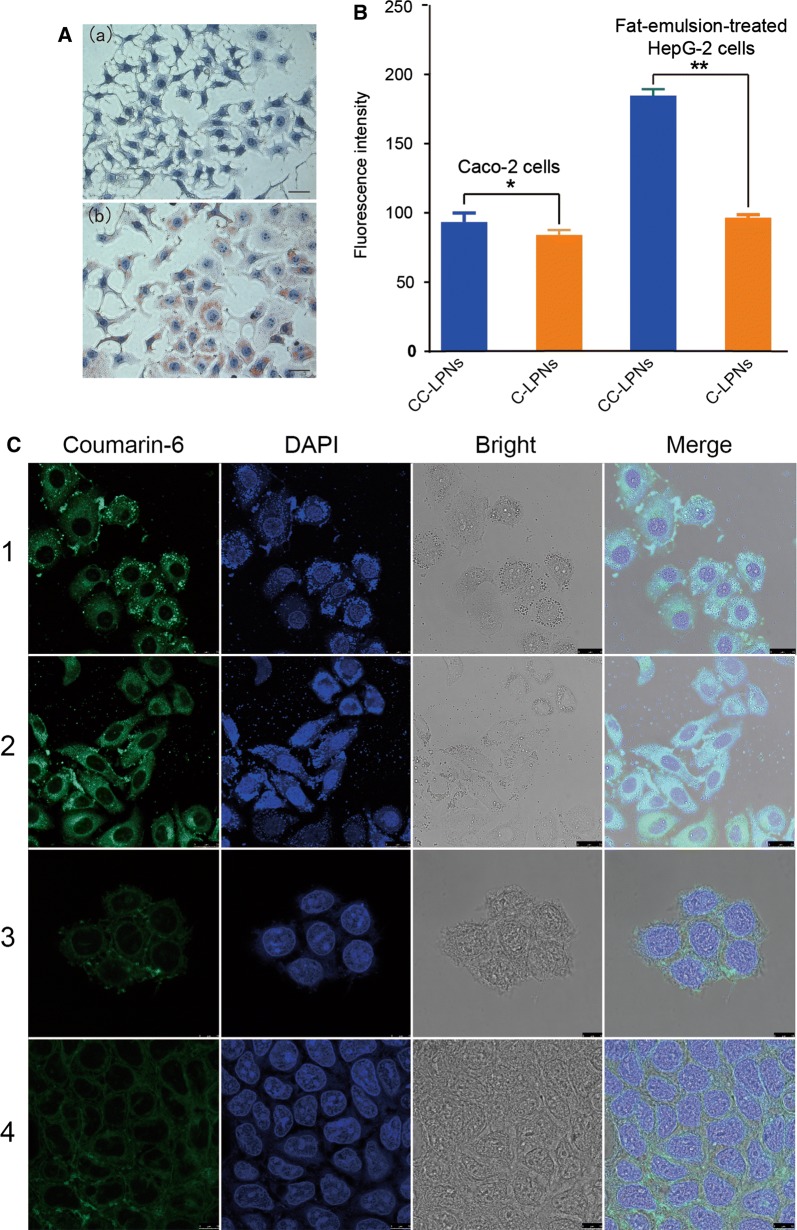
**A** Cell morphology after Oil Red O staining. Left panel, blank control HepG2 cells with no treatment of fat emulsion. Right panel, HepG2 fatty liver cells treated with 1% medical fat emulsion (scale bar, 20 μm). **B** Uptake of nanoparticles by HepG2 fatty liver cells treated with 1% medical fat emulsion. **C** Confocal laser scanning microscopy images. 1. Uptake of curcumin-loaded LPNs (C-LPNs) by HepG2 fatty liver cells treated with 1% medical fat emulsion; 2. Uptake of curcumin-loaded chitosan modified LPNs (CC-LPNs) by HepG2 fatty liver cells treated with 1% medical fat emulsion; 3. Uptake of C-LPNs by Caco-2 cells; 4. Uptake of CC-LPNs by Caco-2 cells

The effects of fat emulsion on lipid accumulation and the morphology of HepG2 cells were evaluated. In Fig. 3A, the orange-red and blue colors indicate neutral fat and nuclei, respectively. The blank HepG2 cells were polygonal with clear edges. In addition, they displayed intercellular tight junctions with no gaps, had large nuclei, and showed few red lipid droplets. HepG2 cells treated with a fat emulsion showed red lipid droplets on the apical side of the cell membrane. In addition, they were round and did not have intercellular tight junctions. Cells with more red lipid droplets had a ring shape formed on the apical side. Taken together, these results indicate that an in vitro lipid deposition model of NAFLD was successfully developed by treatment of HepG2 cells with a fat emulsion, and this model was then used in subsequent cell experiments.

To quantitatively analyze in vitro cellular uptake, nanoparticles were labeled with a fluorescent marker of coumarin-6. Less than 1% of coumarin-6 was released in 1 h. In addition, free coumarin was not directly internalized into cells as reported in a previous study [26]. Therefore, coumarin-6 was used in the cellular uptake study. Furthermore, in this study, coumarin-6-loaded chitosan-modified LPNs (CC-LPNs) and coumarin-6-loaded LPNs (C-LPNs) dispersed in PBS possessing equal fluorescence intensity were used to overcome the influence of self-quenching of coumarin and ensure accuracy in cell uptake assay. Figure 3B shows that the fluorescence intensity in the HepG2 cells treated with fat emulsion was significantly higher after treatment with CC-LPNs than it was after treatment with coumarin-6-loaded LPNs (C-LPNs). Similar results were obtained for the Caco-2 cells. This indicates that CC-LPNs can be taken up by cells. Additionally, chitosan modification enhanced the cellular uptake of the nanoparticles. Confocal microscopy confirmed the uptake of the nanoparticles by Caco-2 cells and fat-emulsion-treated HepG2 cells (Fig. 3C).

### In vitro analysis of intracellular lipid deposition

**Fig. 4:**
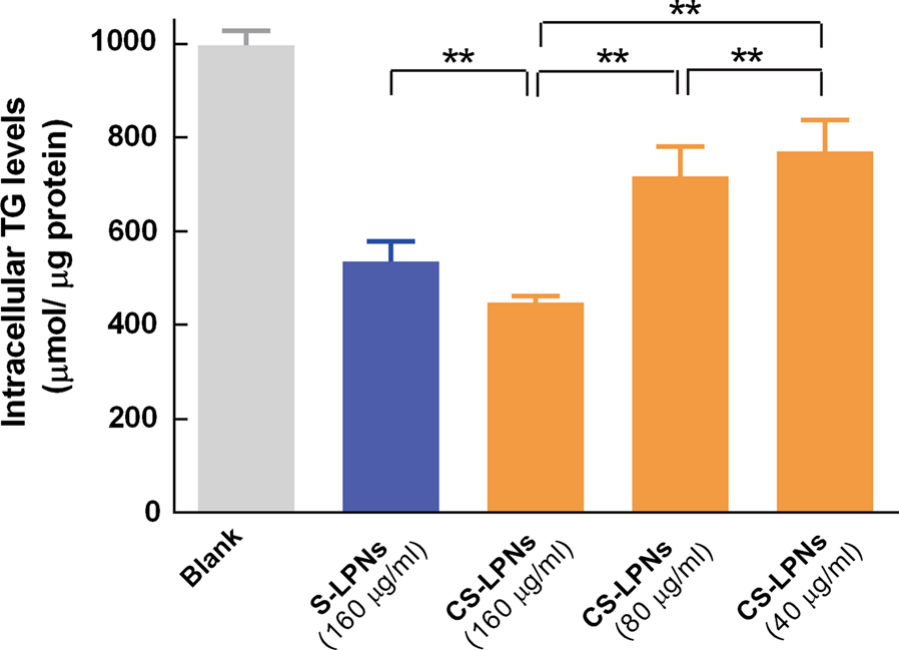
Triglyceride (TG) levels in HepG2 fatty liver cells (n = 3)

The triglyceride content in fat-emulsion-treated HepG2 cells was determined after they were treated with CS-LPNs. As shown in Fig. 4, the CS-LPNs caused a concentration-dependent reduction in triglyceride content. In addition, CS-LPNs caused a much larger decrease in triglyceride content than S-LPNs (*p* < 0.05). These results suggest that CS-LPNs have a beneficial lipid-lowering effect.

### Pharmacokinetic study

**Fig. 5:**
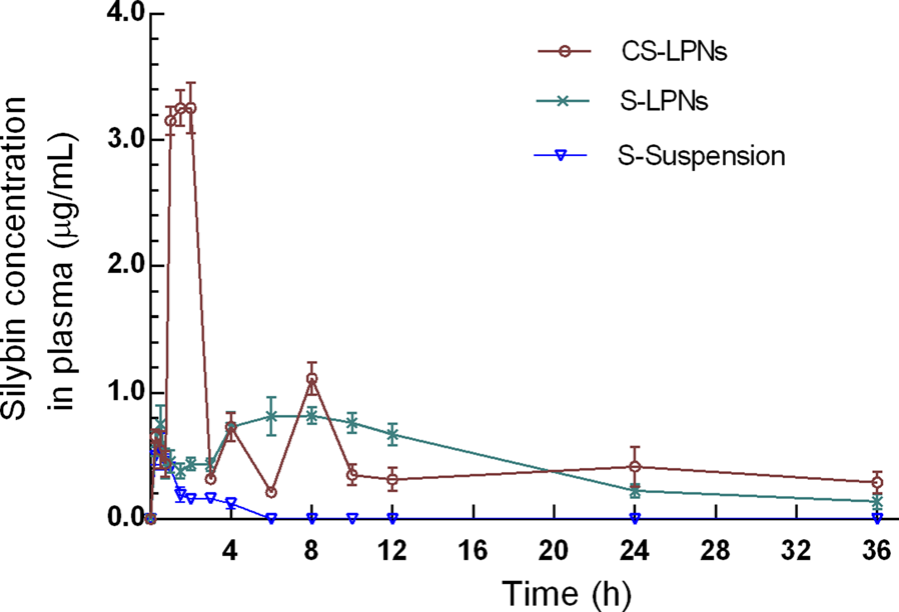
Plasma drug concentration–time curves obtained after oral administration of the various formulations to rats (n = 5)

The plasma concentration–time curves and pharmacokinetic parameters obtained after treating the rats with CS-LPNs, S-LPNs, or silymarin suspension are shown in Fig. 5. The relative bioavailability of CS-LPNs was 1.23-fold and 14.38-fold higher than that of S-LPNs and silymarin suspension, respectively.

### Pharmacodynamic study

**Fig. 6:**
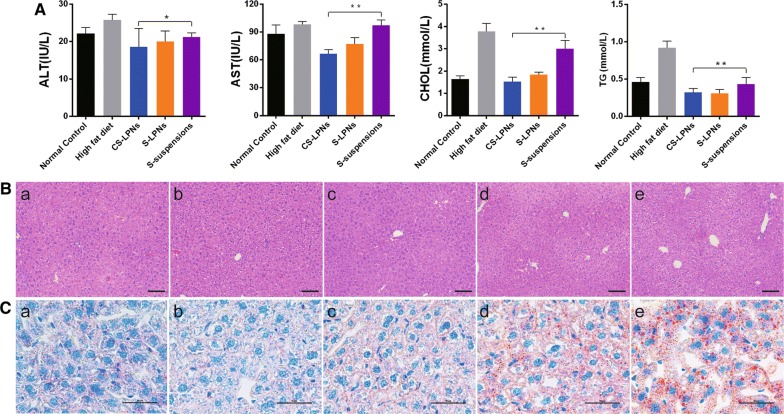
**A** Effects of chitosan-modified silymarin-loaded lipid-polymer hybrid nanoparticles (CS-LPNs) on alanine transaminase (ALT), aspartate transaminase (AST), cholesterol (CHOL), and triglyceride (TG) levels in serum. **B** Hematoxylin and eosin staining results (scale bar, 100 μm). (a) Normal diet, (b) chitosan-modified silymarin loaded-LPNs (CS-LPNs), (c) silymarin-loaded LPNs (S-LPNs), (d) silymarin suspension (S-suspension), (e) high-fat diet (HFD). **C** Oil Red O staining results (scale bar, 50 μm). (a) Normal diet, (b) CS-LPNs, (c) S-LPNs, (d) S-suspension, (e) HFD

PNPLA3 I148M transgenic mice were used to evaluate the hepatoprotective and antihyperlipidemic effects of CS-LPNs in NAFLD. Changes in serum lipid level and liver function indices are shown in Fig. 6A.

Liver function can be indirectly assessed by analyzing serum levels of AST and ALT. As shown in Fig. 6A, serum ALT and AST levels were higher in the HFD group than in the mice that were fed the control diet (normal-diet and control groups). Serum AST and ALT levels were significantly reduced by CS-LPNs when compared to the respective levels in the HFD group (ALT, p < 0.05; AST, p < 0.01). H&E staining was performed to investigate histological changes in the livers of the mice. As shown in Fig. 6B, mice in the HFD group had typical abnormal liver tissue structure. In addition, macrovesicular steatosis and ballooning were observed in hepatocytes. Macrovesicular steatosis was notably less severe in CS-LPN-treated mice than it was in the mice that were treated with silymarin suspension. These results suggest that CS-LPNs protect against liver steatosis in NAFLD.

Hepatic lipid deposition is a key issue in NAFLD; therefore, blood lipid levels were determined to evaluate the lipid-lowering effect of the CS-LPNs. Serum TG and TC levels were higher in the HFD group than in the normal-diet group. In addition, serum TG and TC levels in the CS-LPN-treated group were significantly lower than they were in mice that were treated with silymarin suspension (TC, p < 0.01; TG, p < 0.01). This indicates that the CS-LPNs exert more pronounced antihyperlipidemic and hepatoprotective effects than silymarin alone.

Similar findings were obtained from morphometric analysis of liver sections with ORO staining. In the hyperlipidemia model group, hepatocyte volume increased, steatosis occurred, a large number of red-stained lipid droplets appeared in the cytoplasm, and the nucleus was pushed to the periphery. Furthermore, a remarkable decrease in the number of lipid droplets was observed in the CS-LPN-treated mice, indicating that lipid accumulation in the liver was suppressed. These results suggest that the CS-LPNs exerted a lipid-lowering effect in the model mice.

## Discussion

The main aim of this study was to investigate the potential of chitosan-functioned LPNs as carriers for oral delivery of silymarin, as well as their therapeutic efficiency in an animal model of NAFLD. The LPNs had a negative surface charge due to the presence of lipids on their surface. This enabled easy formation of CS-LPNs via polyionic complex formation between positively charged chitosan and negatively charged LPNs. The physicochemical properties of CS-LPNs, as well as their capacity to encapsulate and release silymarin, were investigated. The change in zeta potential from negative values for LPNs to positive values for CS-LPNs occurred because chitosan is a cationic polysaccharide. In addition, the dynamic light scattering results and TEM images showed that the size of the nanoparticles increased after chitosan coating. The changes in zeta potential and particle size indicated that coating of the particles with chitosan was successful. More importantly, the encapsulation efficiency of silymarin in the CS-LPNs was very high (97.1%). Silymarin-loaded LPNs were composed of PLGA, soybean lecithin, DSPE-PEG2000, and silymarin. Hydrophobic PLGA forms the core of the nanoparticles and allows the encapsulation of hydrophobic silymarin (silybin, LogP 2.8). In addition, the lipid monolayer at the surface of the core provides a molecular fence to reduce silymarin leakage, which is beneficial to enhance the encapsulation efficiency. Similar to other studies, the burst release of silymarin from the LPNs may be attributed to surface-associated drug. However, this release behavior in the first 8 h was slightly alleviated after coating of the particles with chitosan.

Although the cellular uptake of LPNs was generally sufficient, the effect of the chitosan coating was worthy of investigation. Therefore, we compared the uptake of C-LPNs with that of CC-LPNs in Caco-2 cells and fat-emulsion-treated HepG2 cells. As shown in Fig. 3, the cellular uptake of CC-LPNs was higher than that of C-LPNs in both cell lines. However, it was reported that the larger particle size of polymer nanoparticles led to decreased cellular uptake [27]. Although it is not clear whether the larger particle size of CC-LPNs with chitosan coating would result in reduced cellular uptake, it should be noted that the cellular uptake of nanoparticles is influenced by various factors [28, 29]. The cellular uptake results in the present study are consistent with those of a previous study [30]. The positive charge on the chitosan-coated nanoparticles is considered to account for their higher cellular uptake. Further studies are necessary to investigate the involvement of other mechanisms that may have contributed to the improved cellular uptake of the CC-LPNs.

Regarding oral bioavailability, similar to previous investigations, CS-LPNs improved the bioavailability of silymarin compared with silymarin suspensions and chitosan-free LPNs. Similar to other oral nanocarriers, the components of the LPNs may have enhanced encapsulation of hydrophobic silymarin within the particles. This may have improved the solubility of silymarin and enhanced the stability of the formulation in the gastrointestinal tract, which might have facilitated drug absorption by intestinal cells. Additionally, the presence of chitosan on the surface of the nanoparticles may have promoted intestinal absorption by increasing non-specific adhesion to the intestinal mucosa. Furthermore, the CS-LPNs might have undergone improved enterocyte endocytosis. However, compared with other semi-synthetic or synthetic chitosans that would provide better solubility in the gastrointestinal tract, the natural chitosan used in this study seems to be less advantageous, as the bioavailability of CS-LPNs was only 1.23-fold higher than that of the chitosan-free LPNs, although we were not solely interested in bioavailability enhancement. Given the attractive properties of chitosan in terms of hepaprotective effects, we also wanted to investigate whether chitosan thereof was beneficial for therapy of NAFLD by CS-LPNs.

For this purpose, in vitro and in vivo models of NAFLD were used to evaluate the efficacy of the CS-LPNs. Genome-wide association studies have revealed that PNPLA3 I148M polymorphism contributes to differences in lipid levels in the liver and susceptibility to NAFLD [31, 32]. I148M single nucleotide polymorphism (SNP) influences the amount of liver fat. Furthermore, it has been reported that I148M SNP is associated with steatosis and histological severity [33, 34]. Therefore, it is rational to use PNPLA3 I148M transgenic mice as a model to investigate the efficacy and hepatoprotective effect of CS-LPNs in NAFLD. To the best of our knowledge, only a few studies have attempted to explore the potential effects of oral LPNs on NAFLD using PNPLA3 I148M transgenic mice. As we expected, the results of the current study demonstrate that CS-LPNs have a much stronger hepatoprotective effect than chitosan-free LPNs and suspensions. This was evidenced by the changes that the formulation induced in liver histology and serum ALT and AST levels. In addition, the CS-LPNs significantly reduced the total cholesterol concentration, which would result in alleviation of lipid accumulation in the livers of the mice.

The following may have contributed to the enhanced therapeutic effect of the CS-LPNs. In addition to the LPNs yielding a longer duration of drug action in vivo as mentioned above, it has been reported that chitosan itself may be beneficial in the alleviation of NAFLD [35, 36] through promotion of expression of proteins that have roles in hepatic lipid metabolism [37] and activation of antioxidant enzymes such as glutathione peroxidase [38]. Therefore, chitosan might have a synergistic effect or an additive effect in NAFLD treatment in this study. As mentioned above, the in vivo results confirmed that the therapeutic efficiency of CS-LPNs in PNPLA3 I148M transgenic mice was superior to that of silymarin LPNs alone. To further reveal the factors leading to the higher therapeutic effect of CS-LPNs and analyze the contribution of each factor to the overall action, the biological response of chitosan-modified LPNs without silymarin should be studied in future, and thereby the role of chitosan in this case as well as their mechanism in promoting the therapeutic effect in NAFLD may be clearly understood.

In this study, we focused on the effect of CS-LPNs on lipid levels. The pharmacological role of silymarin in NAFLD involves effects on oxidative stress, insulin resistance, and mitochondrial dysfunction [7]. Therefore, the impact of the CS-LPNs on other pathways should be further investigated.

## Conclusion

In the present study, the rational development and utilization of CS-LPNs as effective therapeutics for NAFLD were investigated. CS-LPNs enhanced the uptake of the nanocarriers by fat-emulsion-treated HepG2 cells and Caco-2 cells. This suggests that improved uptake of the nanoparticles could be achieved in vivo, which may increase the oral bioavailability of silymarin. Using a transgenic mouse model of NAFLD, we confirmed that CS-LPNs inhibit lipid accumulation in the mouse liver and enhance the therapeutic efficacy of silymarin. These findings indicate that CS-LPNs may be a new treatment option for NAFLD.

## Materials and methods

### Materials

Poly(lactic-co-glycolic acid) (PLGA) and silymarin (purity > 98%) were purchased from Dalian Meilun Biotechnology Co. Ltd (Dalian, China). Chitosan (75–85% deacetylation, 20–300 cP, mol. wt. 50–190 kDa) and coumarin-6 were purchased from Sigma Aldrich (St. Louis, MO). 1,2-distearoyl-sn-glycero-3-phosphoethanol-amine-*N*-methoxy (polyethylene glycol)-2000 (DSPE-PEG2000) was purchased from A.V.T. Pharmaceutical Co. Ltd (Shanghai, China). Caco-2 cells and HepG2 cells were from Shanghai Institutes for Biological Sciences (Shanghai, China).

### Preparation and characterization of CS-LPNs

CS-LPNs were prepared in two steps. First, LPNs were prepared using a previously reported nanoprecipitation method [39]. Soybean lecithin and DSPE-PEG 2000 were dissolved in ethanol, after which the mixture was dispersed in deionized water at 65 °C under gentle stirring to form a homogenous dispersion (the aqueous phase). The organic phase was composed of poly(lactic-co-glycolic acid) (PLGA) and silymarin dissolved in the acetonitrile-methanol mixture. The organic phase was mixed with the aqueous phase under gentle stirring for 2 h. After removal of the organic solvent by rotary evaporation, the mixture was filtered through a 0.8 μm membrane. Free drug was removed by centrifugal ultrafiltration at 3000 rpm for 5 min. CS-LPNs were formed by incubating the LPN dispersion with an equal volume of chitosan solution (1 mg/mL) for 2 h at 25 °C. The chitosan solution was obtained by dissolving chitosan (molecular weight, 50–190 kDa; degree of acetylation, 15–25%) in 0.5% (v/v) acetic acid, followed by filtration through a 0.8-μm membrane.

Particle size and zeta potential were determined using Zetasizer Nano ZS90 (Malvern Instruments, Malvern, UK). Encapsulation efficiency was calculated by the amount of drug incorporated into the nanoparticles over the initial total loading amount of drug [40]. An ultrafiltration method was used to determine the free amount of silymarin in S-LPNs or CS-LPNs. Briefly, nanoparticles were introduced into a centrifugal filter tube (molecular weight cut-off, 30 kDa, Millipore, Ireland) and centrifuged at 1500*g* for 5 min, followed by detection of the amount of free silymarin by high-performance liquid chromatography (HPLC). To determine the loaded silymarin in nanoparticles, the nanoparticles were destroyed by acetonitrile under ultrasonic treatment for 20 min in ice-water baths. After filtration through 0.22 µm filter, the content of silymarin in the filtrate was analyzed by HPLC. X-ray powder diffraction (XRD) analysis was conducted under the following conditions: radiation source, Cu/Kα; working voltage, 40 kV; current, 100 mA; scanning range, 2θ from 30 to 50 °C; and scanning speed, 80 rpm. DSC measurements were performed on a NETZSCH DSC 204 apparatus (NETZSCH Gerätebau GmbH, Selb, Germany). The scan rate was 10 °C/min with the temperature range of 30 °C–300 °C. Heating was conducted under nitrogen flow.

### In vitro drug release study

In vitro release of silymarin from CS-LPNs was evaluated using the dialysis bag method. The CS-LPNs were dispersed in preheated phosphate-buffered saline (PBS; pH 7.4, 37 °C) containing 0.2% Tween 80, after which the mixture was stirred at 100 rpm for 0.5, 1, 2, 4, 8, 12, 24, or 36 h. Samples (1 mL) were withdrawn at various times to assay drug content by high-performance liquid chromatography (HPLC). Each withdrawn sample was replaced with an equal volume of PBS. The amount of silybin released at each time point was determined, and a graph of percentage release against time was plotted.

### Cellular uptake of the nanoparticles

Caco-2 cells and HepG2 cells were cultured in high-glucose Dulbecco’s modified Eagle’s medium (DMEM) containing 10% fetal bovine serum and 1% penicillin–streptomycin at 37 °C under a 5% CO_2_ atmosphere. The medium was changed every other day. The HepG2 cells were rendered steatotic as previously described [41]. Briefly, when cells reached 50–70% confluence, the medium was replaced with 20% fat emulsion (medium and long chain fat emulsion injection (C8–24); Guangzhou Baxter Qiaoguang Healthcare Co. Ltd., Guangzhou, China) diluted 20-fold with a low-glucose medium. Cellular neutral lipid droplet accumulation in HepG2 cells was measured by Oil Red O (ORO) assay. Briefly, cells were rinsed with PBS (pH 7.4) three times, followed by fixation with cell ORO fixative reagent for 15 min. Next, the cells were rinsed three times and stained with ORO solution for 15 min at room temperature. The cells were then washed sequentially with 60% isopropanol and PBS to remove unbound dye. Nuclei were counterstained with hematoxylin solution for 1 min. After washing, the cells were observed under a microscope.

Cellular uptake of the nanoparticles was investigated in both Caco-2 cells and steatotic HepG2 cells induced by fat emulsion. Chitosan-modified coumarin-6-loaded LPNs (CC-LPNs) were prepared using the method described above except that silymarin was replaced with coumarin-6. The cells were seeded into a 6-well plate at a density of approximately 5 × 10^5^ cells/well, followed by incubation overnight. Next, the culture medium was replaced with a medium containing CC-LPNs (1:1, v/v), after which the cells were incubated for 1 h. The cells were rinsed three times with PBS. After trypsinization and centrifugation, the cells were resuspended in PBS (pH 7.4), followed by lysis and ultrasonication. Acetonitrile was added to the mixture for demulsification, followed by sonication and centrifugation at 12,000 rpm for 10 min. The supernatant was collected and then analyzed at excitation and emission wavelengths of 485 and 528 nm, respectively, using a microplate reader. Fluorescence intensity was measured using a fluorescence microplate reader (Synergy HT; BioTek, Winooski, VT, USA) at excitation and emission wavelength of 485 and 528 nm, respectively.

In vitro cellular uptake was observed by confocal laser scanning microscopy. Cells were seeded (5 × 10^4^ cells/well) and cultured overnight. The cells were washed with PBS three times, incubated with CC-LPNs for 1 h, and rinsed again with PBS three times. After fixation using 4% paraformaldehyde, nuclei were stained with 4′,6-diamidino-2-phenylindole solution. The cells were washed with PBS three times before observation using a TCS SP8 confocal system (Leica, Mannheim, Germany) at excitation wavelengths of 405 nm (blue) and 488 nm (green).

### Quantification of cellular triglyceride content by enzymatic method

Fat-emulsion-treated steatotic HepG2 cells were rinsed with PBS six times to remove the fat emulsion. A suspension of CS-LPNs was mixed with an equal volume of high-glucose DMEM and 1 mL of the resulting mixture was added to the 6-well plate. The cells were incubated at 37 °C for 24 h, followed by rinsing three times with PBS. Next, 600 μL of cell lysis buffer was added to each well. The cells were scraped off with a cell scraper, transferred into a centrifuge tube, and vortexed and lysed for 20 s three times. Samples were allowed to stand at room temperature for 10 min. Next, the centrifuge tubes containing cells were heated at 70 °C for 10 min to denature protein. The cells were then centrifuged at 2,000 rpm for 5 min at room temperature. The supernatant was collected for enzymatic analysis. Triacylglycerol content was determined using a triglyceride assay kit (E1013, for tissues and cells) and a protein quantification kit (bicinchoninic acid) (Applygen Technologies, Beijing, China) according to the manufacturer’s instructions.

### Pharmacokinetic study

Healthy male Wistar rats weighing 250 ± 30 g were randomly divided into CS-LPN, silymarin-loaded LPN (S-LPN), and silymarin suspension groups (n = 6 per group). The silymarin suspension was obtained by dispersing silymarin in polyethylene glycol (PEG) solution (PEG 400 in deionized water, 50:50, v/v). The rats were fasted overnight (12 h) prior to dosing; however, they were allowed free access to water throughout the experiment. The animal experiments were performed in accordance with international guidelines. All experimental protocols were approved by the animal ethics committee of the Institutional Animal Care and Use Committee, Shanghai University of Traditional Chinese Medicine. Each preparation (suspensions, S-LPNs, and CS-LPNs) was administered to rats at a single oral dose of 20 mg/kg (calculated according to the concentration of silybin) by gavage (through a bulb tipped gavage needle that was passed along the esophagus into the stomach). The total volume of the administered preparation was 2 mL. At 60, 90, 120, 180, 240, 360, 480, and 720 min after treatment, 0.5 mL of venous blood was collected into heparinized tubes from the posterior-orbital venous plexus. Each blood sample was centrifuged at 3500 rpm for 5 min to yield plasma, which was frozen at − 80 °C until analysis. Briefly, 1 mL of ethyl acetate was added to plasma, after which the mixture was vortexed for 5 min and centrifuged at 12,000 rpm for 10 min. The organic phase was collected and dried using flowing nitrogen gas. The residue was reconstituted in 200 μL of methanol, vortexed for 3 min, sonicated for 1 min, and centrifuged at 12,000 rpm for 10 min. The supernatant was then analyzed by HPLC. Drug concentrations in plasma were calculated at each time point and a blood concentration–time curve was plotted for each formulation. Pharmacokinetic parameters were analyzed using WinNonlin 6.0 software (Certara, Princeton, NJ, USA).

### Pharmacodynamic study

Specific-pathogen-free (SPF)-grade PNPLA3 I148M transgenic mice (male and female, 5 weeks old; Department of Gastroenterology, Qingdao Municipal Hospital, Qingdao, China) were used for the study. The mice were randomly divided into the following five groups (n = 5 mice per group): CS-LPN, S-LPN, silymarin, hyperlipidemia model, and blank control. Each group was treated daily with the corresponding formulation containing 30 mg/kg silymarin. Mice in the hyperlipidemia model and blank control groups were administered the same volume of 0.9% NaCl solution. The animals were housed in an SPF animal facility under controlled conditions (temperature, 23 ± 2 °C; humidity, 50–70%). Mice in the control group were fed a normal diet, whereas mice in the remaining groups were fed HFD (45% non-alcoholic fatty liver rat diet, HFD group). After 4 weeks, the mice were fasted overnight (12 h) and euthanized with 3.5% chloral hydrate by intraperitoneal injection. The eyeballs were removed, and blood samples were collected. Fresh liver tissues were quickly dissected and frozen, whereas other liver sections were fixed in 4% formaldehyde. Hematoxylin and eosin (H&E) and ORO staining was performed to evaluate lipid accumulation in the liver.

### Serum biochemistry tests

Blood samples were allowed to stand at room temperature for 2 h, followed by centrifugation at 1500*g* for 10 min to obtain serum. The levels of triglycerides (TGs), total cholesterol (TC), high-density lipoprotein cholesterol (HDL-C), low-density lipoprotein cholesterol (LDL-C), alanine transaminase (ALT), and aspartate transaminase (AST) in serum were measured using an automatic biochemical analyzer (Hitachi 7080; Hitachi, Ltd., Tokyo, Japan) by using commercial kits followed by colorimetric measurement.

### Histopathological examination

The liver tissues of PNPLA3 I148M transgenic mice were fixed in 4% formaldehyde, embedded in paraffin for 24 h. The liver sections were placed on microscope glass slides and then stained with hematoxylin and eosin (H&E). Pathological changes in hepatic tissues, such as steatosis and inflammatory cell infiltration, were observed under a light microscope and photographed (Nikon Eclipse Ti-E, Tokyo, Japan).

### Statistical analysis

SPSS 21.0 statistical software (IBM SPSS, Armonk, NY, USA) was used to process the data. Data are presented as the mean ± standard deviation. A *t*-test was used to compare two groups. One-way analysis of variance was used to compare multiple groups. The Student–Newman–Keuls method was used for multiple comparisons between groups. *p* < 0.05 was considered statistically significant.

## Additional file

**Additional file 1: Figure S1.** Characterization of S-LPNs and CS-LPNs. **Figure S2.** Stability results of LPNs and C-LPNs.

## Abbreviations

ALT: alanine transaminase
AST: aspartate transaminase
AUC: area under the plasma silymarin concentration–time curve
CC-LPN: chitosan-modified coumarin-6-loaded lipid-polymer hybrid nanoparticle
C-LPN: coumarin-6-loaded LPN
CS-LPN: chitosan-modified silymarin-loaded LPN
H&E: hematoxylin and eosin
HDL-C: high-density lipoprotein cholesterol
HFD: high-fat diet
HPLC: high-performance liquid chromatography
LDL-C: low-density lipoprotein cholesterol
LPN: lipid-polymer hybrid nanoparticle
NAFLD: non-alcoholic fatty liver disease
ORO: Oil Red O
PBS: phosphate-buffered saline
PEG: polyethylene glycol
PLGA: poly(lactic-co-glycolic acid)
PNPLA3: patatin-like phospholipase-3
S-LPN: silymarin-loaded LPN
SNP: single nucleotide polymorphism
SPF: specific-pathogen-free
TC: total cholesterol
TEM: transmission electron microscopy
TG: triglyceride
XRD: X-ray powder diffraction
