# Supplementary material for: Chitosan-functionalized lipid-polymer hybrid nanoparticles for oral delivery of silymarin and enhanced lipid-lowering effect in NAFLD
Source: J Nanobiotechnology. 2018 Sep 4;16:64. doi: 10.1186/s12951-018-0391-9 (PMC6122632; doi:10.1186/s12951-018-0391-9)
Supplement: Supplementary file 1 — Additional file 1: Figure S1. Characterization of S-LPNs and CS-LPNs. Figure S2. Stability results of LPNs and C-LPNs. [file 12951_2018_391_MOESM1_ESM.docx]

Additional file 1

**Chitosan-functionalized Lipid-Polymer Hybrid Nanoparticles for Oral Delivery of Silymarin and Enhanced Lipid-Lowering Effect in NAFLD**

Jun Liang^1^, Ying Liu^1^, Jinguang Liu, Zhe Li, Qiangyuan Fan, Zifei Jiang, Fei Yan, Zhi Wang, Peiwen Huang, Nianping Feng*

School of Pharmacy, Shanghai University of Traditional Chinese Medicine，Shanghai 201203, China

*Corresponding author:

Nianping Feng (npfeng@hotmail.com; npfeng@shutcm.edu.cn)

**1.Characterization of S-LPNs and CS-LPNs**

Particle size distribution and zeta potential distribution for S-LPNs and CS-LPNs were shown in Figure S1.

**
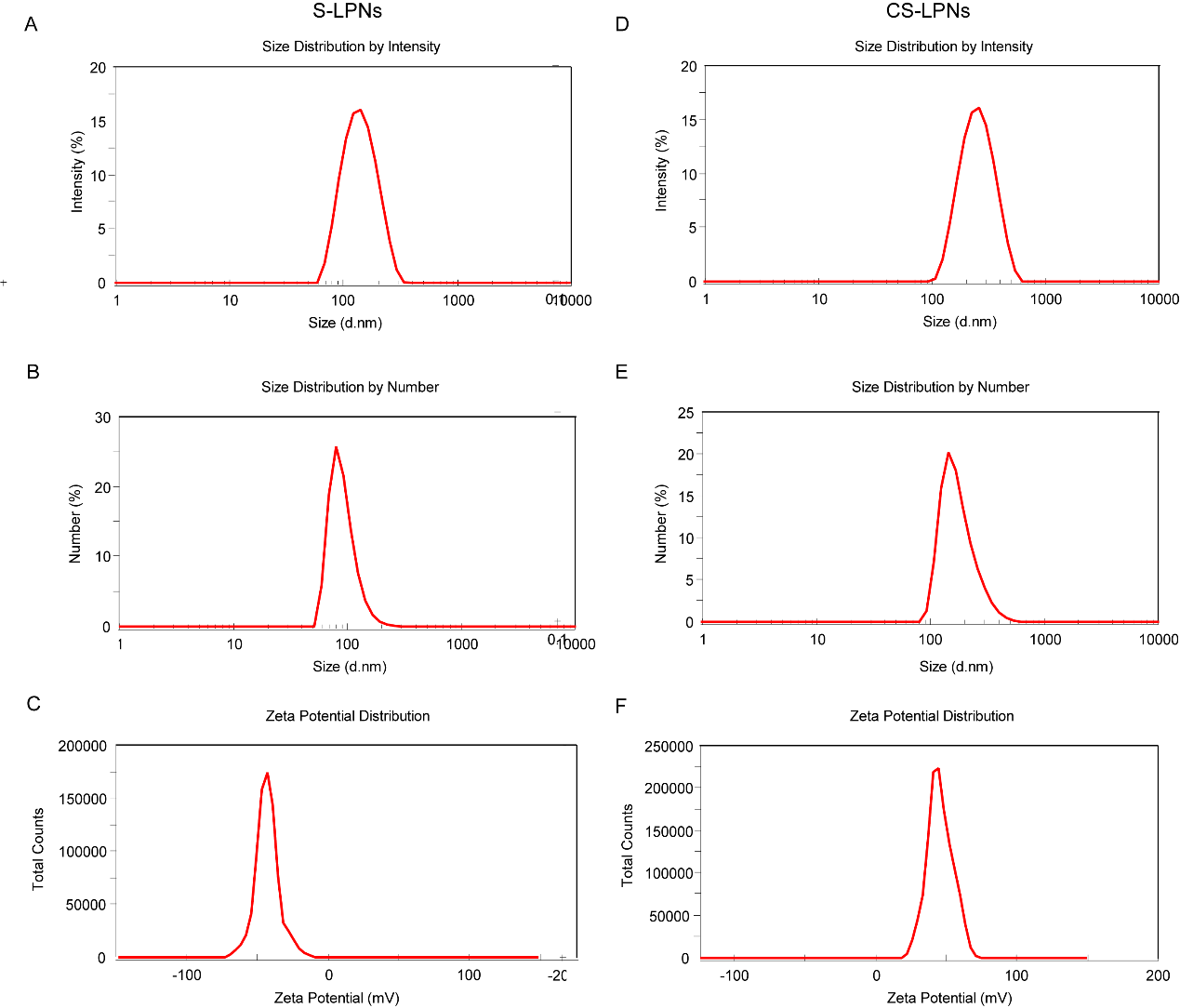
**

Figure S1. Characterization of S-LPNs: Size distribution by intensity (A) and number (B); (C)Zeta potential distribution; Characterization of CS-LPNs: Size distribution by intensity (D) and number (E); (F)Zeta potential distribution.

**2.Stability of Chitosan modified LPNs (C-LPNs) in simulated gastric fluid (SGF) or simulated intestinal fluid (SIF)**

The stability of C-LPNs in SGF and SIF was studied according to the previous report with some modification [1]. In brief, after dispersion of C-LPNs (1 mL) in 50 mL of SGF or SIF, 1 ml of the dispersion was withdrawn at predetermined time point, and the particle size was immediately determined by Zetasizer Nano ZS90 (Malvern Instruments, Malvern, UK).


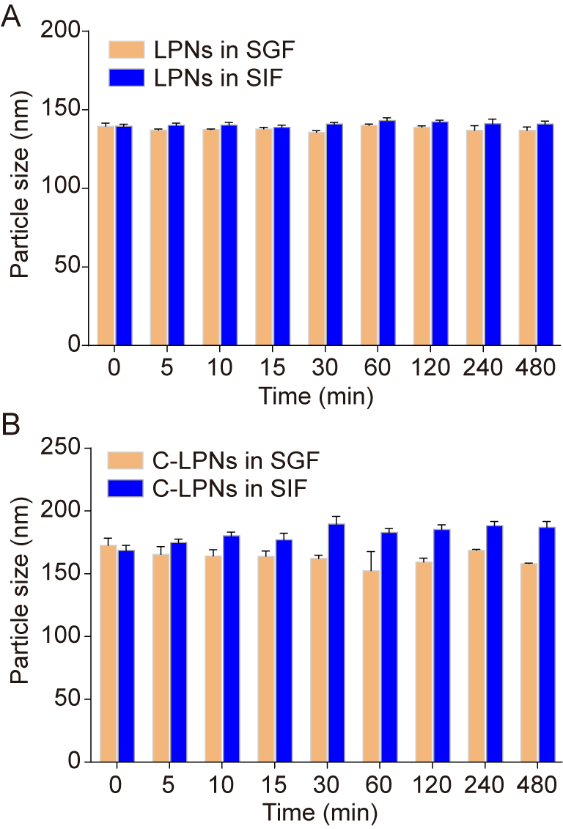


Figure S2. Stability results of LPNs (A) and C-LPNs (B) in SGF and SIF, respectively. (n=3)

As shown in Figure S2, the particle size of LPNs in SGF and SIF kept constant during 480 min, indicating the desirable stability of LPNs in both media. Compared with LPNs, CS-LPNs showed slightly variation in particle size both in SGF and SIF, respectively. The particle size decreased from 172 nm (at time 0) to 158 nm (at time 480 min) in SGF and increased from 169 nm (at time 0) to 187 nm (at time 480 min) in SIF. The changes in hydrodynamic diameters may be caused by the coverage of chitosan. Chitosan is well dissolved in SGF, and part of chitosan might transfer from nanoparticle surface to the surrounding aqueous media until a partition equilibrium was achieved, which might attribute to the decreased particle size. On the contrary, at higher pH value, aggregation of chitosan molecules might occur, leading to the increased in particle size. Taken together, due to limited variation in particle size, the C-LPNs seem to remain stability and integrity in this study.
